# Congenital Trigger Finger

**Published:** 2014-07-10

**Authors:** Sajad Ahmad Salati

**Affiliations:** Department of Surgery, College of Medicine, Qassim University, Saudi Arabia

**Dear Sir**

A 4-month-old boy, first in birth order, born by spontaneous vaginal birth at full term, was brought with a complaint of flexion deformity of the left ring finger. His mother stated that the deformity had been noticed by the primary care physician during routine neonatal check-up at birth. There was no history of peri-natal trauma, infections, or rheumatologic / metabolic disorders and development milestones had been achieved normally. No prior intervention had been done for the complaint. On physical examination, there was triggering of left ring finger (4th digit) with a snap on flexion to about 60° (Fig 1 A and B). Palpation revealed a non-tender, firm nodule over the palmar side of the metacarpophalangeal joints of the affected finger. There was no other anomaly. Plain radiographs of both hands were normal. Parents were counselled and attached to the services of hand physiotherapy/orthotics division where a nocturnal splint was advised with passive daytime, home exercises of finger flexion/extension. However, due to lack of compliance, no benefit could be appreciated after follow up at 8 weeks of presentation. Hence, after informed consent, surgical release of A-1 pulley was undertaken under general anaesthesia and proximal tourniquet control (Fig. 1C). The deformity was markedly reduced after surgical release (Fig. 1D), but the final outcome could not be ascertained due to loss of patient to follow-up. 

Trigger finger (TF) is a rare disorder in paediatric age group [1] though TF is relatively common but the two represent distinct conditions [2]. TF tend to develop earlier in life than trigger thumb and the spontaneous recovery rate was higher in former. The aetiology has been suggested to be genetic though other identifiable causes may be occasionally present [3] and it is still controversial as to whether the congenital trigger digit is truly congenital or an early acquired condition [4]. TF may affect one or more digits [3] and usually affects one hand but bilateral cases are rarely reported in literature [1, 4].

The first line of management is conservative with dedicated home based physiotherapy and splintage (generally nocturnal) to maintain the IP joint in neutral extension or hyperextension [5]. Most of the cases respond. In case of failed conservative management, surgical exploration of flexor mechanism is undertaken to release A-1 pulley, with or without resection of one or both limbs of the sublimis tendon or an A-3 pulley release [6]. A-2 pulley release may be required in extremely rare situations when clinical examination and exploratory findings reveal triggering at A-2 pulley [7]. In our case, our conservative line of management failed primarily due to non-compliance on the part of guardians. The non-compliance remained the issue, even after the surgical release and hence, proper counselling of the guardians is stressed to ensure their cooperation in achieving successful outcome.

**Figure:**
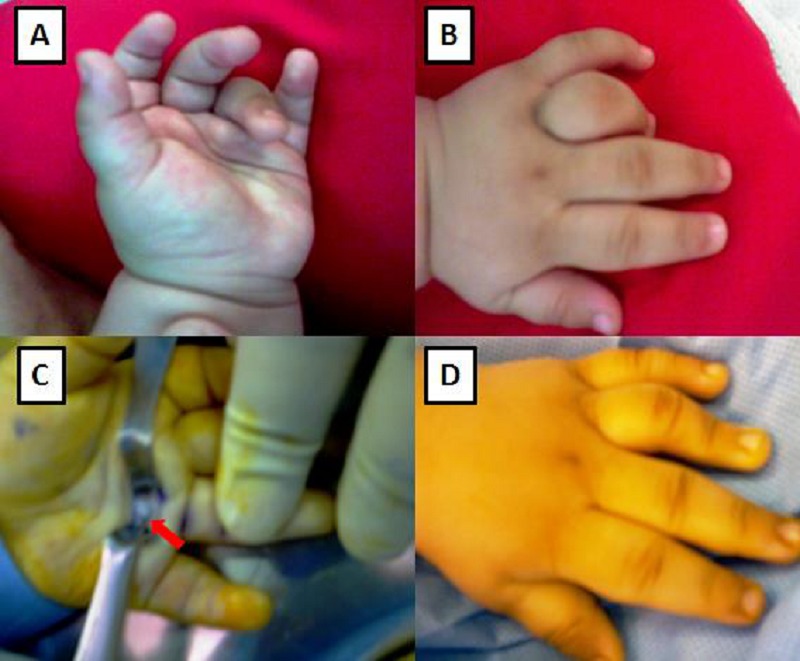
Figure 1 : Congenital trigger left ring finger (A) Palmar aspect (B) Dorsal aspect (C) Surgical release of A-1 pulley (red arrow) (D) Improvement as seen in immediate post-operative period.

## Footnotes

**Source of Support:** Nil

**Conflict of Interest:** None

